# Three-Dimensional Path Planning for UAV Based on Multi-Strategy Dream Optimization Algorithm

**DOI:** 10.3390/biomimetics10080551

**Published:** 2025-08-21

**Authors:** Xingyu Yang, Shiwei Zhao, Wei Gao, Peifeng Li, Zhe Feng, Lijing Li, Tongyao Jia, Xuejun Wang

**Affiliations:** 1 School of Information Science and Technology, Shijiazhuang Tiedao University, Shijiazhuang 050043, China; 2 Hebei Key Laboratory of Electromagnetic Environmental Effects and Information Processing, Shijiazhuang 050043, China; 3 Department of Information Management, Hebei General Hospital, Shijiazhuang 050051, China; 4 Department of Mechanical and Electrical Engineering, Shijiazhuang Information Engineering Vocational College, Shijiazhuang 052161, China; 5 Hebei Provincial Engineering Research Center for Vertical Take-Off and Landing Fixed-Wing Intelligent Unmanned Aerial Vehicle Technology and Applications, Shijiazhuang 052161, China

**Keywords:** UAV path planning, intelligent control, dream optimization algorithm, optimal path

## Abstract

The multi-strategy optimized dream optimization algorithm (MSDOA) is proposed to address the challenges of inadequate search capability, slow convergence, and susceptibility to local optima in intelligent optimization algorithms applied to UAV three-dimensional path planning, aiming to enhance the global search efficiency and accuracy of UAV path planning algorithms in 3D environments. First, the algorithm utilizes Bernoulli chaotic mapping for population initialization to widen individual search ranges and enhance population diversity. Subsequently, an adaptive perturbation mechanism is incorporated during the exploration phase along with a lens imaging reverse learning strategy to update the population, thereby improving the exploration ability and accelerating convergence while mitigating premature convergence. Lastly, an Adaptive Individual-level Mixed Strategy (AIMS) is developed to conduct a more flexible search process and enhance the algorithm’s global search capability. The performance of the algorithm is evaluated through simulation experiments using the CEC2017 benchmark test functions. The results indicate that the proposed algorithm achieves superior optimization accuracy, faster convergence speed, and enhanced robustness compared to other swarm intelligence algorithms. Specifically, MSDOA ranks first on 28 out of 29 benchmark functions in the CEC2017 test suite, demonstrating its outstanding global search capability and conver-gence performance. Furthermore, UAV path planning simulation experiments conducted across multiple scenario models show that MSDOA exhibits stronger adaptability to complex three-dimensional environments. In the most challenging scenario, compared to the standard DOA, MSDOA reduces the best cost function fitness by 9% and decreases the average cost function fitness by 12%, thereby generating more efficient, smoother, and higher-quality flight paths.

## 1. Introduction

In recent years, unmanned aerial vehicles (UAVs) have been widely applied with the maturation of technology due to their advantages such as small size, light weight, strong adaptability, high concealment, and low risk factors [1,2]. The application scenarios of UAVs are constantly expanding in multiple fields, from military reconnaissance, disaster monitoring, agricultural spraying, to logistics transportation [3,4,5,6]. The autonomous flight capability and exceptional mission execution efficiency of UAVs make them a key tool in numerous industries [7,8,9]. However, as the application scope continues to expand, the challenges faced by UAVs are gradually increasing, especially the issue of path planning in complex environments [10,11]. Therefore, how to achieve efficient, safe, and accurate flight path planning in a complex environment [12] has become one of the core issues in the research of unmanned aerial vehicle technology [13].

UAV path planning algorithms can be divided into two main types: traditional algorithms and intelligent optimization algorithms. The first category is traditional algorithms, including the A* algorithm [14], Dijkstra algorithm [15], artificial potential field method [16], rapid search random tree [17], etc. The second method is a meta-heuristic algorithm [18]. Unlike traditional algorithms that rely on specific problem models, meta-heuristic algorithms employ heuristic rules and strategies to globally search for the optimal or approximate optimal solution to a given problem [19]. Therefore, the research of UAV path planning has shifted from traditional algorithms to meta-heuristic algorithms [20]. Meta-heuristic algorithms are algorithms that solve complex problems by imitating some optimization processes in nature or society [21]. In UAV path planning, meta-heuristics can effectively deal with uncertainty, complex environments, or dynamically changing conditions [22]. Numerous meta-heuristic algorithms, including particle swarm optimization (PSO) [23,24], Ant Colony Optimization (ACO) [25,26], Grey Wolf Optimization (GWO) [27,28], Sparrow Search Algorithm (SSA) [29,30], and Harris hawks optimization (HHO) [31,32], have been successfully applied to the problem of UAV path planning. While these algorithms have demonstrated robust performance across a range of optimization problems, their application to UAV path planning still faces challenges, such as limited convergence accuracy and susceptibility to local optima.

Researchers have proposed various improved schemes to address the challenges of meta-heuristic algorithms in UAV path planning. Xiao et al. [33] employed a Logistic chaotic map initialization and the Nutcracker Optimization Algorithm to enhance the quality of the initial population. Wang et al. [34] combined Tent chaotic mapping and Gaussian mutation strategy to solve the slow convergence speed and the easy fall into local optimization problems of the traditional BKA algorithm in high-dimensional data and complex function optimization. Zhou [35] et al. introduced a nonlinear control mechanism to optimize the convergence factor of the GWO algorithm, which improved the adaptability and robustness of the algorithm. Hu et al. [36] proposed a co-evolutionary multi-group particle swarm optimization (CMPSO), which innovatively improves the global optimization ability by introducing two different group learning mechanisms and a grouping mechanism based on the activity level to avoid convergence to local optima. Zhang et al. [37] introduced the Cauchy mutation strategy and adaptive weights into the search process and combined them with the Sine–cosine Algorithm (SCA) to improve the global search ability and convergence efficiency. Xu et al. [38] combined the whale optimization algorithm with the dung beetle optimization algorithm to improve local search ability.

The dream optimization algorithm (DOA) proposed by Gao et al. [39] in 2024 is a novel intelligent optimization algorithm inspired by the characteristics of human dreaming. The algorithm incorporates a basic memory strategy, a forgetting and replenishing strategy that balances exploration and exploitation, and a dream sharing strategy to enhance the ability to escape local optima. The optimization process is divided into two phases: exploration and exploitation. DOA exhibits advantages such as fast convergence speed, strong stability, and high optimization precision, making it suitable for complex engineering problems. However, despite its rapid convergence, the algorithm is prone to becoming trapped in local optima, and its original mechanism tends to rapidly lose population diversity, leading to a poor ability to escape local optima. This paper proposes a multi-strategy dream optimization algorithm (MSDOA), with the following key contributions:

1. A population initialization method using the Bernoulli chaotic map is employed to initialize the population, enhancing the diversity of the initial population, promoting a more even distribution across the entire search space, and expanding the coverage range.
2. The proposed adaptive hybrid perturbation mechanism dynamically adjusts disturbance parameters by combining Cauchy variation and Lévy flight strategies during the forgetting and supplementing phases of the dream process. This approach enhances the ability to explore the solution space while preserving high local search accuracy, thereby accelerating convergence.
3. To evade local optima, a lens-imaging learning strategy is employed during the exploration phase. This approach simulates the symmetric mapping of individuals in the search space to produce “mirror image” solutions, thereby improving the ability to escape local traps.
4. This study presents a new global perturbation mechanism, Adaptive Individual-level Mixed Strategy (AIMIS), aimed at improving global optimization performance. AIMIS combines two individual-level perturbation strategies: a global perturbation that utilizes boundary information to expand the search space and a local perturbation that leverages variances among individuals to enhance precision.

The remainder of this paper is organized as follows: Section 2 presents the problem formulation of UAV path planning. Section 3 introduces the standard dream optimization algorithm. Section 4 outlines the technical details of the proposed MSDOA. Section 5 provides comparative experimental results and analysis between MSDOA and other state-of-the-art intelligent optimization algorithms. Section 6 summarizes the main conclusions of this work.

## 2. Problem Description of UAV Path Planning

Examining UAV path planning difficulties involves creating an extensive cost function comprising flight path length, threat, altitude, and smoothness costs, with various constraint weights. The calculation of the cost function is as follows:

### 2.1. Flight Path Length Cost

**Figure 1:**
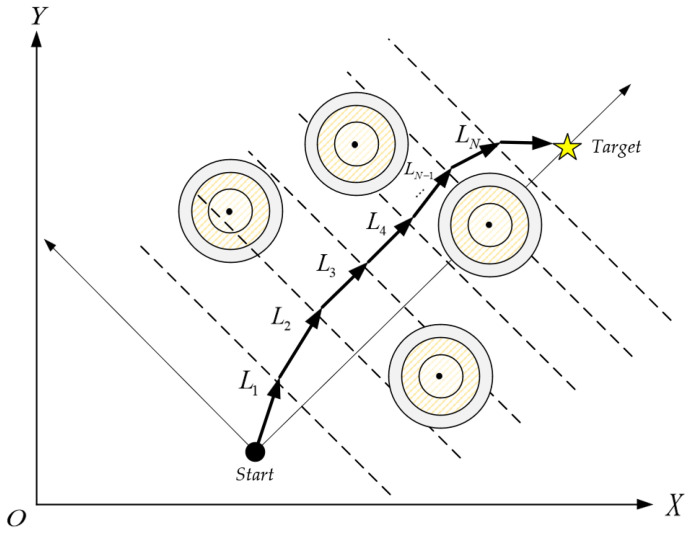
Top-down view of flight path.

The flight path length cost of the UAV reflects the distance from the starting point to the destination. The coordinates of each waypoint are represented as $L_{ij}=\left(x_{ij}{,y}_{ij}{,z}_{ij}\right)$, and the Euclidean distance between two waypoints is calculated as $\left\|\vec{L_{ij}L_{i,j+1}}\right\|$. A top view of the UAV flight path is shown in Figure 1. The fight path length cost *F_distance_* is mathematically modeled as shown in Equation (1).

(1) $$F_{distance}=\sum_{j=1}^{n-1}\left\|\vec{L_{ij}L_{i,j+1}}\right\|$$

where *L_ij_* and *L_i_*_,*j*+1_ represent the *j* and *j* + 1 path points in the *i*th flight path.

### 2.2. Threat Cost

**Figure 2:**
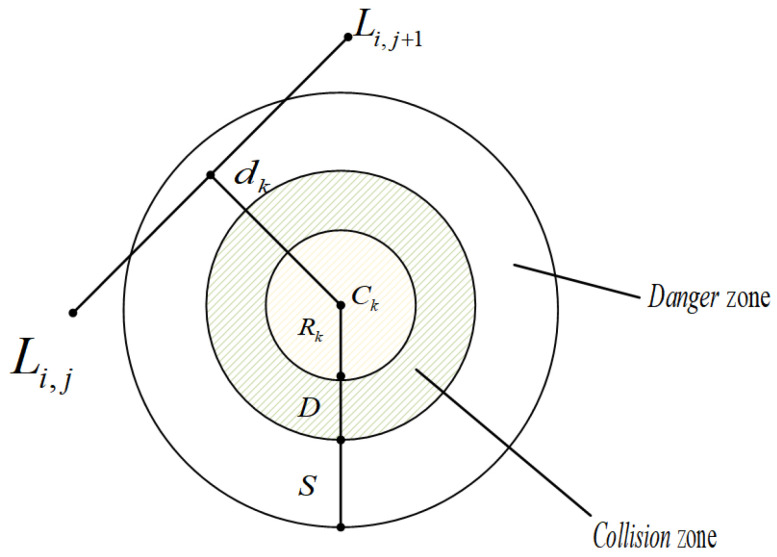
Threat prediction map.

In order for the UAV to reach the set target safely, ensuring that the UAV does not collide with obstacles is the main requirement. In this paper, it is assumed that each threat is a cylinder, and the entire flight area is divided into a safety area, threat area, and collision area. Figure 2 is a threat prediction diagram, which illustrates the relationship between these three areas. Threat cost *F_threat_* is expressed by Equation (2).

(2) $$F_{threat}=\sum_{j=1}^{n-1}\sum_{k=1}^{K}T_{k}(\vec{L_{ij}L_{i,j+1}})$$

where $T_{k}(\vec{L_{ij}L_{i,j+1}})$ represents the threat cost function for the path segment $\vec{L_{ij}L_{i,j+1}}$ with respect to the *k*-th threat, as defined in Equation (3).

(3) $$T_{k}(\vec{L_{ij}L_{i,j+1}})=\left\{\begin{array}{l} \infty \;if\;d_{k}\leq D+R_{k}\\ (S+D+R_{k})-d_{k}\;if\;D+R_{k}<d_{k}\leq S+D+R_{k}\\ 0\;if\;d_{k}>D+S+R_{k}\; \end{array}\right.$$

where *k* represents the quantity of obstacles present. *C_k_* denotes the center of each obstacle, while *R_k_* signifies the radius of the obstacle. *D* stands for the diameter of the UAV, *d_k_* represents the distance between the current UAV position and the center of the *k*-th obstacle, whereas *S* indicates the critical safety distance.

### 2.3. Flight Altitude Cost

**Figure 3:**
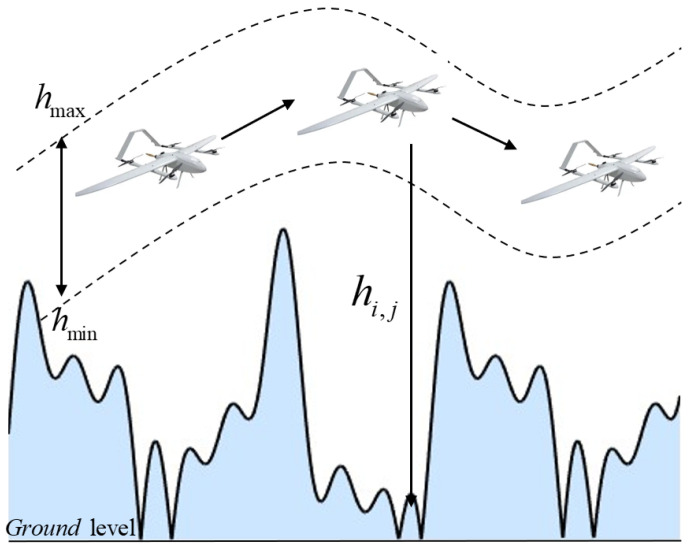
Flight height constraint diagram.

When UAVs fly at high altitudes, they are more susceptible to external environmental influences, which increases the risk of flight accidents. Conversely, flying at excessively low altitudes poses the danger of ground collisions. Therefore, the altitude cost *F_altitude_* for height-constrained flight trajectories at the maximum and minimum altitudes is expressed by Equation (4). Figure 3 illustrates the altitude constraint concept, showing the allowable flight corridor between $h_{\min}$ and $h_{\max}$, as well as the terrain-relative height $h_{ij}$.

(4) $$F_{altitude}=\sum_{j=1}^{n}H_{ij}$$

where *H_ij_* represents the height cost of the current path point, calculated as shown in Equation (5).

(5) $$H_{ij}=\left\{\begin{array}{ll} \left|h_{ij}-\frac{\left(h_{\max}+h_{\min}\right)}{2}\right| & if\;h_{\min}\leq h_{ij}\leq h_{\max}\\ \infty & otherwise \end{array}\right.$$

where *h_max_* and *h_min_* represent the maximum and minimum flight altitudes of the UAV, respectively; *h*_ij_ denotes the altitude above ground level.

### 2.4. Smoothing Cost

Smoothness of the flight path during UAV operations is crucial and is primarily impacted by the yaw and pitch angles. To enhance path smoothness, a smoothing cost is incorporated into the path optimization procedure. This cost aims to minimize the curvature of the flight trajectory and mitigate abrupt variations in the yaw angle.

**Figure 4:**
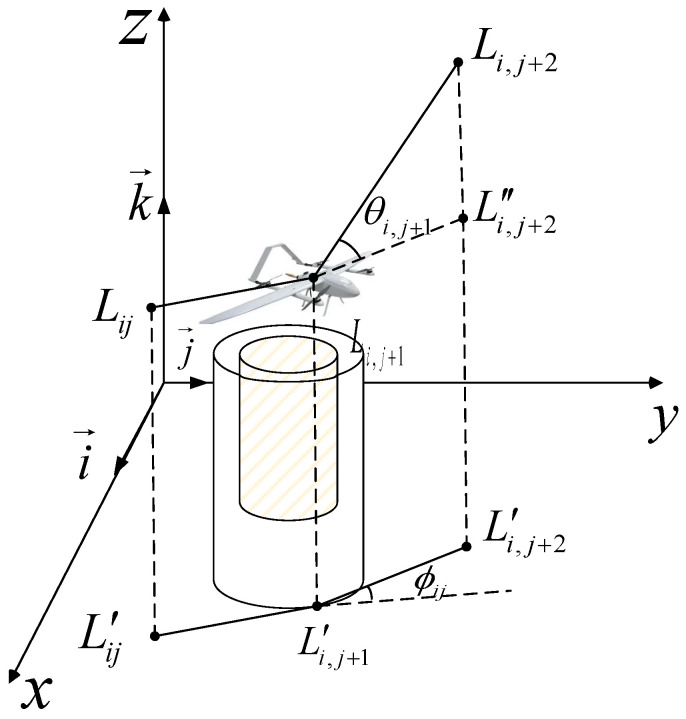
Flight angle diagram.

In Figure 4, the yaw angle *ϕ_ij_* indicates the deviation between vectors $\vec{{L^{\prime }}_{ij}{L^{\prime }}_{i,j+1}}$ and $\vec{{L^{\prime }}_{i,j+1}{L^{\prime }}_{i,j+2}}$, representing two consecutive path segments, while the pitch angle *θ_ij_* signifies the angle between vector $\vec{L_{i,j+1}L_{i,j+2}}$ and its projection on the horizontal plane. Assuming $\vec{k}$ is the unit vector along the *z*-axis, the vector between two consecutive points is determined according to Equation (6). The projection vector, along with the yaw angle *ϕ_ij_* and pitch angle *θ_ij_*, are defined in Equations (7) and (8).

(6) $$\vec{{L^{\prime }}_{ij}{L^{\prime }}_{i,j+1}}=\vec{k}\times (\vec{L_{ij}L_{i,j+1}}\times \vec{k})$$

(7) $$\phi_{ij}=\arccos\left(\frac{\vec{{L^{\prime }}_{ij}{L^{\prime }}_{i,j+1}}\cdot \vec{{L^{\prime }}_{i,j+1}{L^{\prime }}_{i,j+2}}}{\left\|\vec{{L^{\prime }}_{ij}{L^{\prime }}_{i,j+1}}\times \vec{{L^{\prime }}_{i,j+1}{L^{\prime }}_{i,j+2}}\right\|}\right)$$

(8) $$\theta_{ij}=\arctan\left(\frac{z_{i,j+1}-z_{ij}}{\vec{\left\|{L^{\prime }}_{ij}{L^{\prime }}_{i,j+1}\right\|}}\right)$$

Therefore, the smoothing cost function is expressed in Equation (9).

(9) $$F_{smooth}=a_{1}\sum_{j=1}^{n-2}\phi_{ij}+a_{2}\sum_{j=1}^{n-1}\left|\theta_{ij}-\theta_{i,j-1}\right|$$

where *a*_1_ and *a*_2_ represent the penalty coefficients for yaw angle and pitch angle, respectively.

### 2.5. Total Cost Function

The total cost function of traversing a path includes the flight path length cost, threat cost, altitude cost, and smoothing cost. The mathematical model is applicable to general path planning problems. In this study, the optimal flight path of the UAV is determined by minimizing the cost function defined in Equation (10).

(10) $$F_{total}=\omega_{1}\cdot F_{distance}+\omega_{2}\cdot F_{threat}+\omega_{3}\cdot F_{altitude}+\omega_{4}\cdot F_{smooth}$$

where *w*_1_, *w*_2_, *w*_3_, and *w*_4_ are the weighting coefficients for the flight path length cost, threat cost, altitude cost, and smoothing cost, respectively.

Based on the principles of safety and operational efficiency, the weighting coefficients *w*_1_, *w*_2_, *w*_3_, and *w*_4_ are defined as follows:

The highest weight, *w*_3_ = 10, is assigned to the altitude cost. Considering the practical need for high-altitude flight in real-world missions, this indicates that altitude safety is given the highest priority in the path planning process.

*w*_1_ = 5 reflects that the distance cost also carries significant weight, emphasizing that while ensuring safety, it is still important to control the total flight path length to improve operational efficiency.

The lower weight of *w*_2_ = 2 suggests that most threats can be effectively avoided through altitude selection, allowing altitude and distance constraints to primarily guide the path planning process.

Finally, *w*_4_ = 1, although smoothness contributes to the stability of the flight control system, in this configuration, mission safety and execution efficiency are prioritized over path smoothness.

## 3. Standard Dream Optimization Algorithm

The ensuing section delineates the fundamental principles and procedure of the standard DOA, explaining its mathematical model and providing a comprehensive analysis of its mechanisms.

### 3.1. Initialization

The initialization of the population is a crucial step in heuristic, dream-inspired optimization algorithms. The initial population is generated within the search space to initiate the optimization process. The equation for obtaining the initial population is as in Equation (11).

(11) $$X_{i}=X_{l}+rand\times (X_{u}-X_{l}),i=1,2,\ldots ,N$$

where *N* represents the number of individuals in the population, *X_i_* denotes the i-th individual in the population, *X_l_* and *X_u_* indicate the lower and upper boundaries of the search space, and rand is a *D*-dimensional vector where each dimension is a random number between 0 and 1.

### 3.2. Exploration Phase

The exploration phase of the algorithm begins by partitioning the population into five distinct groups based on variations in memory capacity. These differences in memory capability are reflected in the parameter *k_q_*, which represents the number of forgotten dimensions for each group. Prior to the “dreaming” event, a memory strategy is applied, where all individuals in a group observe the best-performing individual from previous iterations. Subsequently, the forgetting and replenishment strategy, as well as the dream-sharing strategy, are executed. Accounting for the disparities in memory capacity among individuals, each individual randomly selects certain information dimensions to forget, referred to as “forgotten dimensions.” During subsequent updates, individuals adjust their positions solely along these marked forgotten dimensions.

#### 3.2.1. Memory Strategies

Dreams are inherently connected to existing memories. Prior to dreaming, group *q* recalled the positions of the optimal individuals within the group and adjusted their own positions accordingly. The update formula for the optimal individual is shown in Equation (12).

(12) $$X_{i}^{t+1}=X_{bestq}^{t}$$

where $X_{i}^{t+1}$ represents the position of the i-th individual at time t + 1, $X_{bestq}^{t}$ denotes the position of the best individual in the *q*-th group at time t, and *q* = 1, 2, 3, 4, 5 indicates the group number.

#### 3.2.2. Forgetting and Supplementation Strategy

The proposed forgetting and supplementation strategy exploits disparities in memory to stochastically discard and replenish specific dimensions, thereby augmenting both global and local search capacities. The position update equation is presented in Equation (13).

(13) $$X_{i,j}^{t+1}=X_{bestq,j}^{t}+(X_{l,j}+rand\times (X_{u,j}-X_{l,j}))\times \frac{1}{2}\times (cos(\pi \times \frac{t+T_{\max}-T_{d}}{T_{\max}})+1)$$

where $X_{i,j}^{t+1}$ denotes the position of the *i*-th individual in the *j*-th dimension at iteration *t* + 1, $X_{bestq,j}^{t}$ represents the position of the best individual in the *q*-th group in the *j*-th dimension at iteration *t*, $X_{u,j}$ and $X_{l,j}$ denote the upper and lower bounds of the search space in the *j*-th dimension, *t* is the current iteration count, $T_{\max}$ is the maximum number of iterations, and $T_{d}$ is the maximum iteration count during the exploration phase.

#### 3.2.3. Dream-Sharing Strategies

The dream-sharing strategy in DOA also follows the memory strategy, allowing individuals to randomly acquire positional information from others in the forgetting dimension. The update formula is represented by Equation (14).

(14) $$X_{i,j}^{t+1}=\left\{\begin{array}{l} X_{m,j}^{t+1}\;m\leq i\\ X_{m,j}^{t}\;i<m\leq N \end{array}\right.{\;j=K}_{1},\;K_{2}\ldots ,\;K_{kq}$$

where *m* is a population randomly selected from 1 to *N* populations.

### 3.3. Exploitation Phase

The Exploitation phase no longer involves further grouping. Instead, the optimal solution from the exploration phase is selected, and the position of each individual within the forgetting dimension is subsequently updated. This updating process follows a similar approach to Equations (12) and (13). Equation (15) shows that in dimensions other than *K*_1_, *K*_2_, …, *K_k_*, individuals retain the position of the global best solution from previous iterations, effectively preserving this information during the dreaming phase. In contrast, Equation (16) illustrates that in dimensions *K*_1_, *K*_2_, …, *K_k_*, individuals discard this information and instead regenerate new positions through self-organization, as updated as follows by Equations (15) and (16).

#### 3.3.1. Memory Strategies

(15) $$X_{i}^{t+1}=X_{best}^{t}$$

where $X_{i}^{t+1}$ denotes the *i*th individual at iteration t + 1; $X_{best}^{t}$ represents the best individual of the whole population at iteration *t*.

#### 3.3.2. Forgetting and Supplementation Strategy

(16) $$X_{i,j}^{t+1}=X_{best,j}^{t}+(X_{l,j}+rand\times (X_{u,j}-X_{l,j}))\times \frac{1}{2}\times (cos(\pi \times \frac{t}{T_{\max}})+1)$$

where $X_{i,j}^{t+1}$ represents the position of the *i*-th individual in the *j*-th dimension at iteration *t* + 1; $X_{best,j}^{t}$ represents the position of the best individual of the entire population in the *j*-th dimension at iteration *t.*

## 4. Multi-Strategy Dream Optimization Algorithm

This section establishes the mathematical model of the multi-strategy dream optimization algorithm based on the analysis of the basic version presented in the previous section. It provides a detailed description of the improved mechanism, presents the corresponding pseudo-code, and flowchart.

### 4.1. Improved Algorithm Based on Bernoulli Chaotic Map

The initial population of the dream optimization algorithm is randomly generated, often resulting in uneven distribution and reduced population diversity. This, in turn, can negatively impact the algorithm’s convergence speed. To address this issue, a chaos mapping mechanism is employed to enhance population diversity and improve algorithmic efficiency. The nonlinear and periodic properties of chaos mapping enable the generation of more complex and effective search results. Specifically, the Logistic, Tent, and Bernoulli chaotic maps are utilized for population initialization. Compared to other maps, the Bernoulli chaotic map exhibits a wider search range and can generate initial solutions more uniformly. The Bernoulli chaotic map [40] initialization produces a more uniform sequence than random initialization, facilitating faster searches for optimal solutions and mitigating the risk of becoming trapped in local minima.

The present work employs the Bernoulli chaotic map to initialize the population, a strategy that yields a more uniform population distribution and rapidly generates initial path points exhibiting strong randomness. The obtained values are then projected into the chaotic variable space using the Bernoulli mapping relationship. The specific expression of this mapping is presented in Equation (17).

(17) $$x(t+1)=\left\{\begin{array}{cc} \frac{x(t)}{1-\alpha } & 0\leq x(t)\leq 1-\alpha \\ \frac{x(t)-(1-\alpha )}{\alpha } & 1-\alpha <x(t)\leq 1 \end{array}\right.$$

where x(t) is a chaotic variable at time *t*, α represents the chaotic component.

The generated chaotic values are then mapped into the initial population of the algorithm through linear transformation, with the mapping formula given in Equation (18).

(18) $$X=X_{l}+x(X_{u}-X_{l})$$

### 4.2. Adaptive Hybrid Perturbation Strategy

During the forgetting and supplementation phase, this paper introduces an adaptive hybrid perturbation mechanism to enhance the coverage of the solution space and further improve search efficiency in this phase. This study proposes an adaptive individual hybrid perturbation strategy. This strategy integrates three perturbation methods:

1. A basic uniform random perturbation to enhance population diversity;
2. A Cauchy mutation [41] factor *C_y_* to leverage its heavy-tailed distribution and improve local escape capabilities, *C_y_* as shown in Equation (19);

(19) $$C_{y}=\frac{1}{2}+\frac{1}{2}\times tan(0.5\cdot \pi (rand-0.5))$$

3. The incorporation of a Lévy flight-based perturbation *RL* [42], which enables long-distance jumps and improves global exploration. The mathematical expression for *RL* is given in Equation (20), as follows:

(20) RL=l×levy(λ)

where *l* represents the step length control parameter. levy(λ) is a path that obeys the Lévy distribution, which represents the introduced Lévy flight strategy.

The selection of these perturbation strategies is governed by an adaptive probability control mechanism, which dynamically adjusts the selection probabilities based on the current iteration progress. This adaptive scheduling enables a smooth transition from broad exploration in the early stages to refined exploitation in the later phases of optimization. μ represents the perturbation term generated according to the adaptive strategy.

Under this mechanism, the update rule for the *i*-th individual and the *j*-th variable at iteration *t* is defined in Equation (21).

(21) $$X_{i,j}^{t+1}=X_{bestq,j}^{t}+(X_{l,j}+\mu \times (X_{u,j}-X_{l,j}))\times \frac{1}{2}\times (cos(\pi \times \frac{t+T_{\max}-T_{d}}{T_{\max}})+1)$$

where μ update formula in the equation is as shown in Equation (22):

(22) $$\mu =\left\{\begin{array}{ll} rand & if\;h_{1}<P_{rand}^{i}\\ C_{y} & if\;h_{1}<P_{rand}^{i}+P_{cauchy}^{i}\\ RL & otherwise \end{array}\right.$$

where *h*_1_ is a uniformly distributed random number, and $P_{rand}^{i}$, $P_{cauchy}^{i}$ and $1-P_{rand}^{i}-P_{cauchy}^{i}$ represent the dynamically adjusted selection probabilities for each perturbation type at iteration *i.*

### 4.3. Lens Imaging Learning Strategy for Population Update

**Figure 5:**
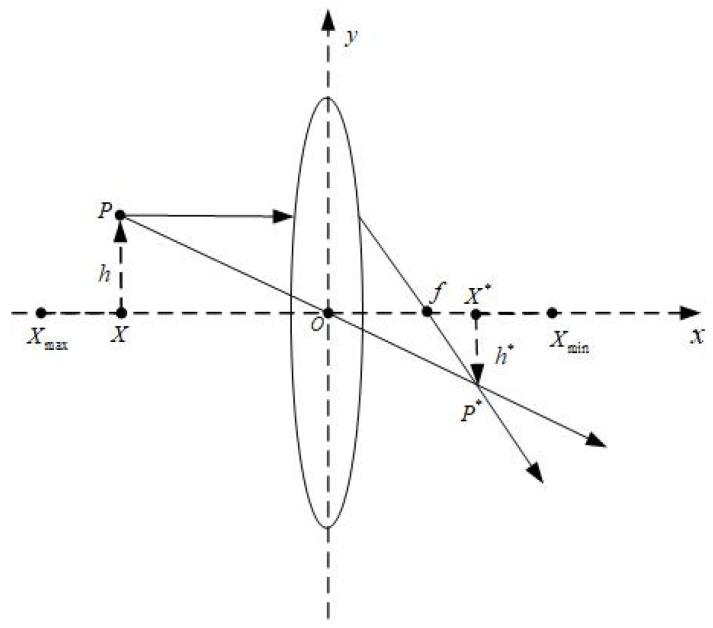
Principle diagram of lens imaging inverse learning.

In the original dream optimization algorithm, the algorithm exhibits strong exploitation capability but is prone to falling into local optima. To address this issue, this paper introduces a lens imaging reverse learning strategy into the population update process. Compared with traditional backward learning strategies, the lens imaging reverse learning incorporates a scaling factor k, as illustrated in Figure 5. By generating the reverse solution of the current solution through inverse operation and comparing its fitness with that of the original solution, the initial solution is updated, thereby further enhancing the algorithm’s probability of escaping local optima. The lens imaging reverse learning strategy is described in Equation (23), and *k* is the scaling coefficient, whose expression is given in Equation (24).

(23) $$X_{i,j}^{*}(t)=\frac{\left(X_{u}+X_{l}\right)}{2}+\frac{\left(X_{u}+X_{l}\right)}{2k}-\frac{X_{i,j}(t)}{k}$$

(24) $$k={\left(1+{\left(t/T\right)}^{0.5}\right)}^{10}$$

where $X_{i,j}^{*}$ is the generated inverse solution, and *X_i_*_,*j*_(t) is the current solution.

### 4.4. Adaptive Individual-Level Mixed Strategy

The proposed Adaptive Individual-level Mixed Strategy (AIMIS) addresses the limitations of the forgetting supplement strategy and dream-sharing strategy in the DOA algorithm. These traditional methods often rely excessively on local perturbations, leading to convergence in local optima and restricting the algorithm’s global search capability. AIMIS integrates two distinct perturbation mechanisms at the individual level to enhance global optimization performance. The first mechanism employs a global perturbation based on boundary information and chaotic sequences, aiming to expand the search scope. The second mechanism utilizes local perturbations based on differential information among individuals, targeting improved search precision. Simultaneously, a greedy selection mechanism is employed to retain individuals with superior fitness from multiple perturbation candidates, ensuring the quality of perturbation operations. Compared to traditional fixed perturbation methods, AIMIS effectively disrupts the local convergence of individuals and reconstructs perturbation paths. This enhances the algorithm’s ability to escape local optima and improves its efficiency and diversity in exploring diverse solution regions within the search space. The key to AIMIS lies in applying appropriate perturbation operations to the population, dispersing it more widely in the solution space, avoiding concentration in certain regions, and increasing the algorithm’s search capability in the global solution space. The mathematical formulation of this strategy is presented in Equation (25).

(25) $$x_{i,j}^{t+1}=\left\{\begin{array}{ll} x_{i,j}^{t}+{\left(1-\frac{t}{T_{\max}}\right)}^{\frac{2}{T_{\max}}}\times (lb+rand\times (ub-lb)\times H) & rand\leq \tau \\ x_{i,j}^{t}+[rand\times (1-rand)+rand]\times (x_{w,j}^{t}-x_{v,j}^{t}) & rand>\tau \end{array}\right.$$

where *H* is a random number of 0 or 1, and $x_{w,j}^{t}$ and $x_{v,j}^{t}$ individuals are randomly selected from the population.

### 4.5. The MSDOA Algorithm Flow

**Figure 6:**
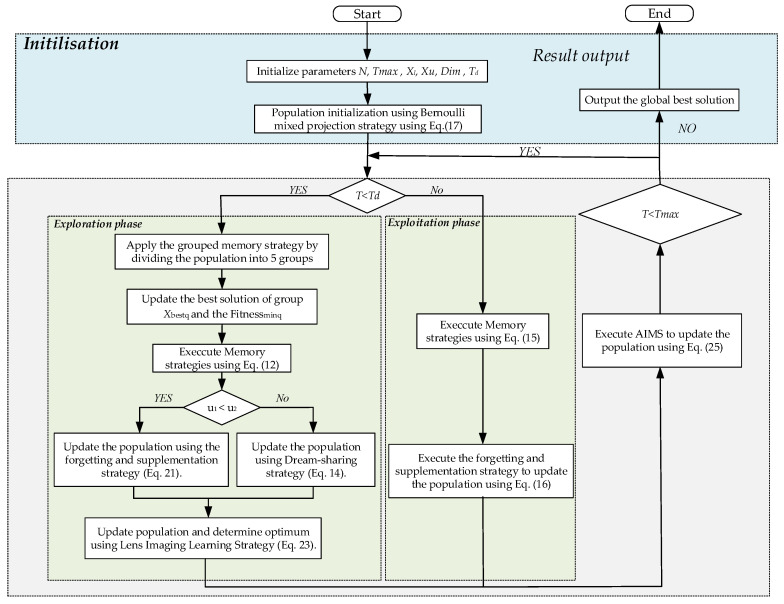
Flowchart of MSDOA.

Combined with the mathematical model of the above-mentioned multi-strategy dream optimization algorithm, Figure 6 presents the overall flowchart of MSDOA, the execution steps of MSDOA can be summarized into the following seven steps:

Step 1: Initialize parameters population size *N*, the maximum number of iterations $T_{\max}$, the lower limits of variables *X_l_*, the upper limits of variables *X_u_*, and the size of the problem Dim. The number of iterations is a demarcation *T_d_*.

Step 2: By means of Equation (17), incorporate the Bernoulli chaotic map to initialize the population.

Step 3: If the current iteration number *t* < *T_d_*, then enter the exploration phase; otherwise, enter the Exploitation phase.

Step 4: During the exploration phase, the group memory strategy is implemented to identify the optimal individual within each group. Two random probabilities, u1 and u2, are then evaluated; the condition u1 < u2 is judged. If u1 < u2 holds true, the adaptive disturbance strategy incorporates the dream forgetting and supplementation; otherwise, the dream sharing strategy is utilized to direct the individual’s search. Following this, the sub-populations are consolidated, and an inverse learning strategy, as per Equation (23), is employed to enhance the search capabilities of the population.

Step 5: In the development stage, the memory strategy is adopted directly, and then the dream forgetting and supplementation strategy are implemented to improve local search ability.

Step 6: At the end of each iteration, Adaptive Individual-level Mixed Strategy updates the population positions according to Equation (25), further avoiding local optima and improving the algorithm’s global search capability.

Step 7: Verify whether the current iteration number equals the maximum Tmax. If so, conclude the process and display the global optimal solution; otherwise, advance to step 3 and proceed with the subsequent iteration.

The detailed procedural steps of MSDOA are further illustrated in the corresponding pseudo-code, as shown in Algorithm 1.
**Algorithm 1:** Pseudo-code of MSDOA**Input:** Initialize parameters *N*, *Tmax*, *X_l_*, *Xu*, *Dim*, *T_d_.***Output:** The global best solution *X*_gbest_ and f(*X*_gbest_)1:Bernoulli chaotic map Initialize population P = {*X*_1,_ *X*_2,…,_ *X_N_*} using Equations(17) and (18).2:while *t* < *Tmax*3:    **while** *t* < *T_d_* **do**4:        Update the best solution *X*_gbest_ and minimum fitness Fitness_min_5:        **for** q = 1: 5 **do**6:                Update the best solution of group *X*_bestq_ and the Fitness_minq_7:                Update *X_i_*_,*j*_ using Equation (12)8:                **for** *i* = (((q − 1)/5 × *N*) + 1:(q/5 × *N*) do9:                        **if**     u_1_ < u_2_ **then**10:                                Update *X_i_*_,*j*_ using Equation (21)11:                                Check the bounds of *X_i_*_,*j*_12:                        **else**13:                                Update *X_i_*_,*j*_ using Equation (14)14:                                Check the bounds of *X_i_*_,*j*_15:                        **end if**16:                        Calculate $X_{i,j}^{*}$ using Equation (23)17:                        **if** f(*X_i_*_,*j*_) > f($X_{i,j}^{*}$) **then** *X_i_*_,*j*_ = $X_{i,j}^{*}$18:                        **else** *X_i_*_,*j*_ = *X_i_*_,*j*_19:                        **end if**20:                **end for**21:        **end for**22:        Update the current number of iteration *t* by *t* = *t*+123:    **end while**24:    **while** *t ≥ T_d_* and *t < Tmax* **do**25:        Update $X_{i}^{t+1}$ using Equation (15)26:        **for** *i* = 1: *N* **do**            Update *X_i_*_,*j*_ using Equation (16)27:                Check the bounds of *X_i_*_,*j*_28:        **end if**29:        Update the current number of iteration *t* by *t* = *t*+130:    **end while**31:    Adaptive Individual-level Mixed Strategy Update $X_{i}^{t+1}$ using Equation (25)32:**end while**

## 5. Simulation and Results Analysis

The present study evaluated the performance of the MSDOA algorithm against a diverse set of widely adopted optimization algorithms, including the classical and well-established particle swarm optimization (PSO) [43], Grey wolf optimizer (GWO) [44], Harris hawks optimization algorithm (HHO) [45], as well as the recently published and highly competitive Crested Porcupine Optimizer (CPO) [46], BKA [47], and Sand Cat swarm optimization (SCSO) [48]. The assessment was conducted using standard benchmark test functions and path planning simulation experiments, examining key metrics such as global search capability, convergence rate, and stability. This comprehensive comparative analysis aimed to elucidate the advantages of the MSDOA approach in addressing complex optimization challenges.

### 5.1. Comparison of Algorithms in the CEC2017 Test Set

To further evaluate the efficacy of the MSDOA algorithm on high-dimensional and large-scale test functions, benchmark functions from the CEC2017 test suite are used for simulation testing. This test suite encompasses a variety of function types, such as single-peak, multi-peak, mixed, and compound functions, exhibiting high complexity. By utilizing this diverse set, the adaptability and optimization efficiency of the algorithm across various problem characteristics. To ensure the robustness and accuracy of our analysis, the maximum number of iterations during the exploration phase was set to *T_d_* = 0.9 × *T_max_*. Standardized testing protocols and datasets were employed, with each test function executed independently 30 times over 500 iterations to minimize the impact of randomness on the results.

The optimization performance of various algorithms was assessed through three key metrics: the Best fitness value (Best), the mean error (Mean), and the standard error (Std). As shown in Table 1, the MSDOA algorithm ranked first among the twenty-eight benchmark test functions and ranked third in the F14 benchmark test, demonstrating that the incorporation of a multi-strategy optimization approach into the basic Dream Algorithm significantly enhanced the optimization accuracy and search speed. Notably, the MSDOA algorithm exhibited significantly superior mean error and standard error values compared to other optimization algorithms, highlighting its exceptional stability, robustness, and overall performance across diverse test cases.

**Figure 7:**
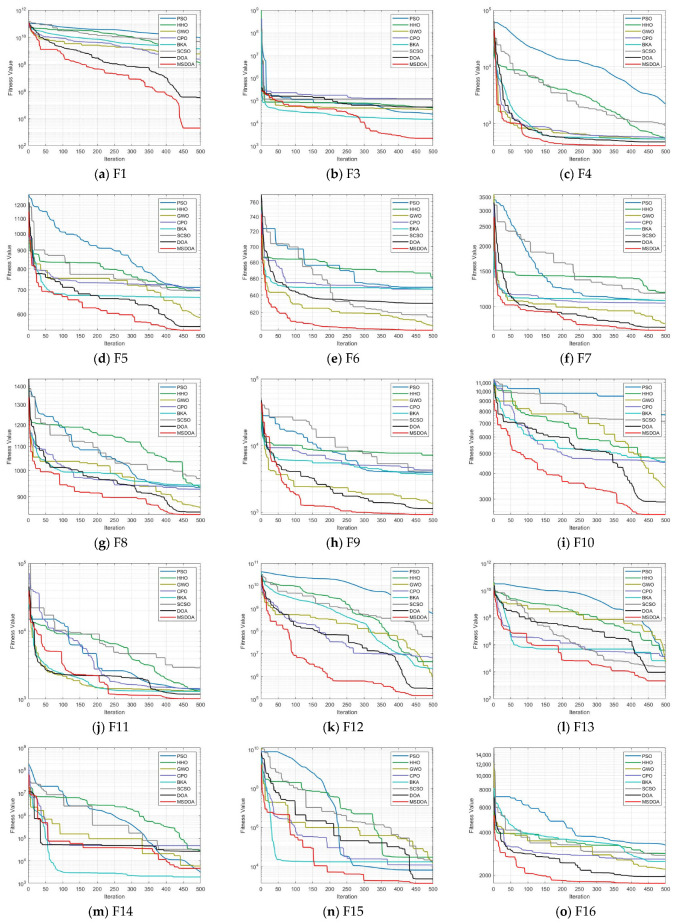

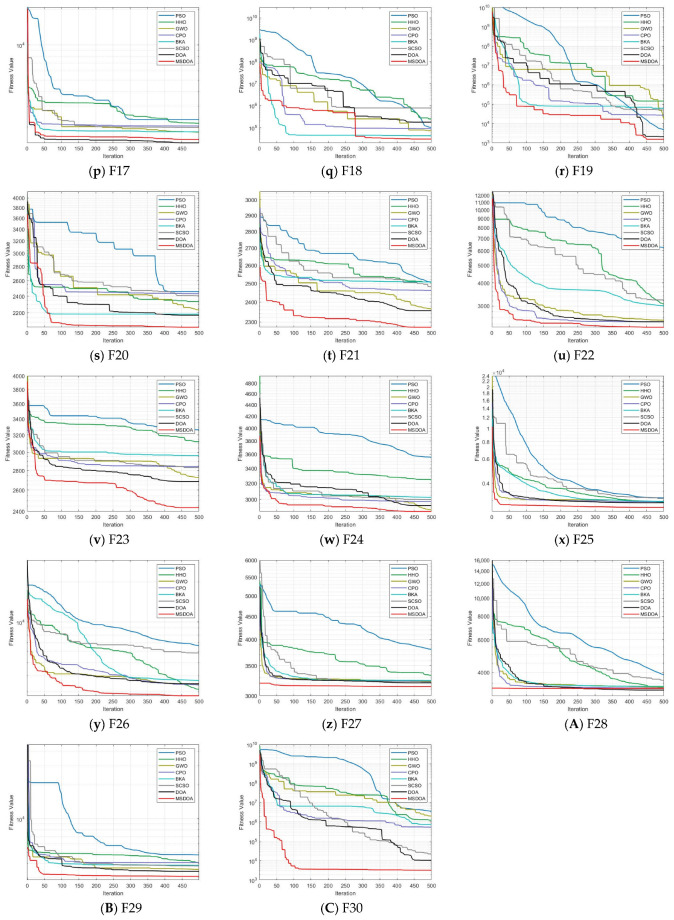
Convergence curve of test function algorithm.

Figure 7 presents the convergence curves for various optimization algorithms across different test functions, with the MSDOA algorithm depicted in red. The MSDOA algorithm demonstrates a rapid decrease in fitness, achieving substantially lower levels at the maximum number of iterations, outperforming other optimization algorithms. This indicates that MSDOA not only swiftly converges to an optimal solution but also surpasses other algorithms in optimization efficiency and accuracy. Specifically, MSDOA shows an extremely fast convergence speed and an extremely low final error in the unimodal functions F1, F3, and F6, indicating that it has good local search accuracy. Among the multimodal functions F5, F7, F9, and F10, it is possible to effectively escape the local optimal trap and maintain a continuous optimization trend. For the complex-structured mixed functions F12, F13, and F20, MSDOA can adaptively adjust the strategy among different function regions to achieve global convergence. However, on the most challenging composite functions F23, F26, and F30, it can still maintain a stable decline and eventually reach an excellent optimal solution level, demonstrating strong robustness and generalization ability.

**Figure 8:**
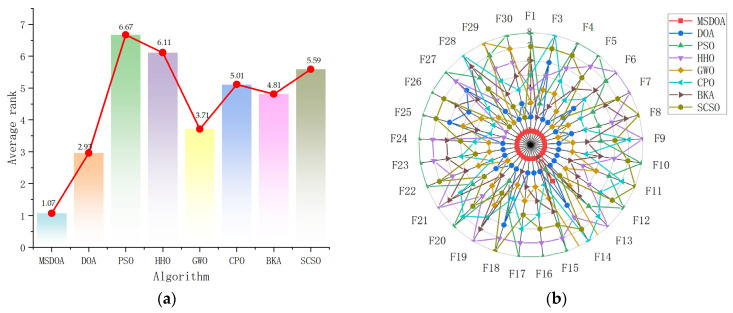
Ranking charts of optimization results on the CEC2017 benchmark. (**a**) The average rank chart. (**b**) The radar chart.

The comprehensive analysis presented in the rank analysis Figure 8a and radar chart Figure 8b conclusively demonstrates the superior performance and remarkable stability of the MSDOA algorithm across the 30 test functions. With an outstanding average ranking of 1.07, MSDOA clearly surpasses all other algorithms, emphasizing its exceptional optimization capabilities. Moreover, the uniform distribution of the radar chart and the centralized vertices further reinforce the algorithm’s excellent stability, highlighting its optimal optimization performance in the CEC2017 benchmark test.

These results clearly demonstrate that MSDOA exhibits strong adaptability, high search efficiency, and robust global optimization capability on the CEC2017 test set, making it well-suited for a wide range of complex real-world optimization problems.

### 5.2. Performance Test and Analysis of UAV Track Planning Under Different Algorithms

#### 5.2.1. Performance Analysis Under Different Obstacles

Three scenarios were developed to evaluate the effectiveness of the algorithm in simulating UAV paths in three-dimensional mountainous terrain. Table 2 provides a detailed overview of the scenes. In each scenario, UAV paths were planned with six trajectory points, navigating through different obstacle densities: three obstacles in scenario 1, six obstacles in scenario 2, and nine obstacles in scenario 3. These scenarios were designed to assess the performance of the MSDOA algorithm in environments of increasing complexity, ranging from simple to highly complex terrains. The minimum flying altitude was established at 100 m, while the maximum altitude was capped at 300 m. Both the maximum turning angle and climbing angle were restricted to 45° to ensure optimal maneuverability and flight safety.

**Figure 9:**
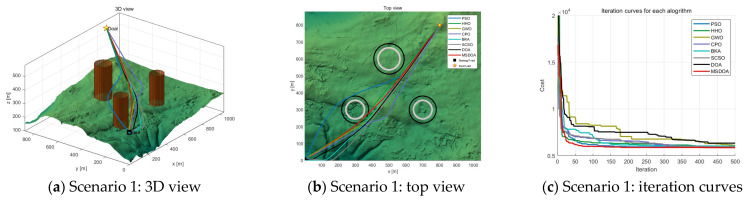
Comparison of path planning results in scenario 1 (number of waypoints = 6).

**Figure 10:**
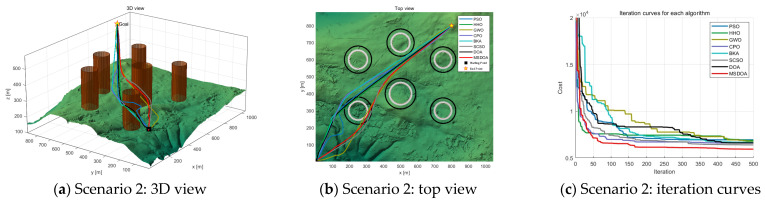
Comparison of path planning results in scenario 2 (number of waypoints = 6).

**Figure 11:**
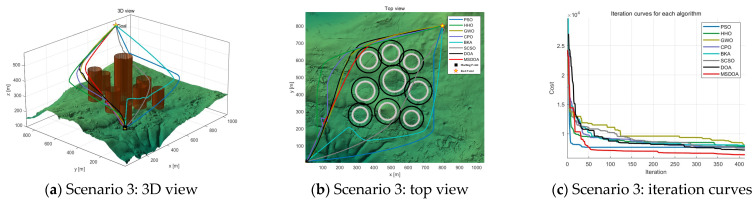
Comparison of path planning results in scenario 3 (number of waypoints = 6).

As the number of obstacles increases, the algorithm’s search difficulty also rises. Figure 9, Figure 10 and Figure 11 illustrate the flight path’s side view, top view, and convergence curve for each scenario, respectively. Table 3 presents the cost function fitness values for each optimization algorithm across scenarios. With heightened environmental complexity, the paths generated by each algorithm become more convoluted, underscoring their adaptability differences. In Figure 9a, within a simple environment, all algorithms successfully avoid obstacles, though PSO and CPO produce more winding paths, the optimal solutions across algorithms show little variance. Notably, as depicted in Figure 9c, MSDOA converges more rapidly at the iteration’s onset, demonstrating superior optimization efficiency and maintaining robust planning capability in simpler environments.

Figure 10 illustrates the planning outcomes in complex environments, showcasing the diverse paths taken by various algorithms and their adaptability fluctuations. The results suggest that most algorithms struggle with accuracy and robustness in high-complexity settings. However, the DOA algorithm stands out by achieving an optimal value of 6410, while the MSDOA algorithm surpasses all others with an impressive optimal value of 5941. Remarkably, MSDOA achieves this optimal value in just around 150 iterations, highlighting its exceptional search efficiency. In Figure 11, the path planning results from different optimization algorithms in highly complex scenarios are depicted. As the environmental complexity increases, the performance gaps between the algorithms become more pronounced. Particularly noteworthy are the paths generated by the BKA and PSO algorithms, which exceed the constraints of the map. Table 3 provides a comparative analysis showing that MSDOA achieves a 7.81% reduction in the cost function fitness compared to DOA by 7.81% and further reduces the cost function by 13.3%, 10.7%, 11.6%, 12.1%, 15.8%, and 8.48% compared to PSO, HHO, GWO, CPO, BKA, and SCSO, respectively. These results are achieved while ensuring rapid and stable convergence across iterations. Additionally, the stability of MSDOA, as indicated by its mean and standard deviation, underscores its robustness and adaptability in handling highly complex tasks.

#### 5.2.2. Performance Analysis with Different Numbers of Waypoints

**Figure 12:**
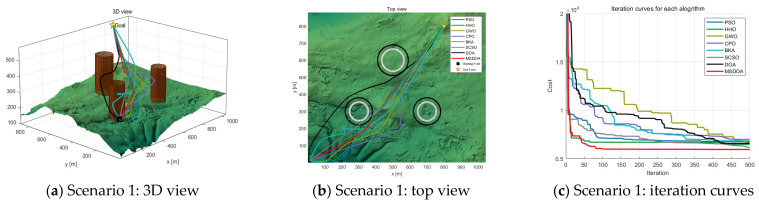
Comparison of path planning results in scenario 1 (number of waypoints = 12).

**Figure 13:**
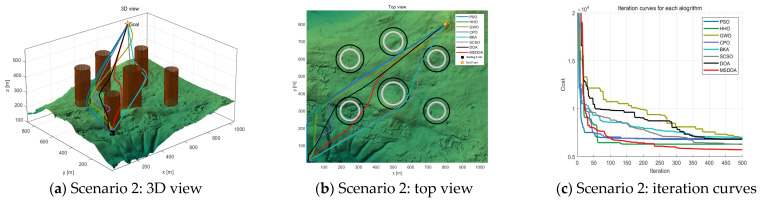
Comparison of path planning results in scenario 2 (number of waypoints = 12).

**Figure 14:**
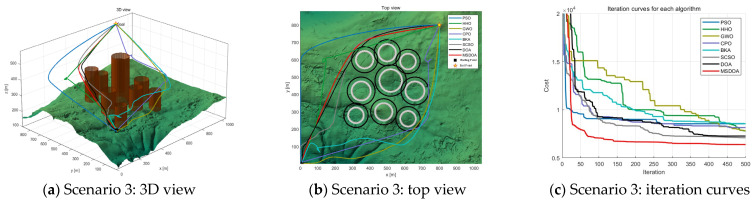
Comparison of path planning results in scenario 3 (number of waypoints = 12).

The number of waypoints has a significant impact on the computational efficiency and trajectory search performance of the algorithm. As the number of waypoints increases from 6 to 12, Table 4 shows the fitness values of the cost function for each optimization algorithm under different scenarios at 12 waypoints. The results indicate that the optimal values of all algorithms are improved, but the MSDOA algorithm demonstrates stronger adaptability and reliability. As illustrated in Figure 12, Figure 13 and Figure 14, the flight paths generated by the algorithms become more tortuous as the number of waypoints increases. In Scenario 1, the MSDOA-planned flight path is smoother than the paths generated by the other algorithms. Furthermore, by comparing the UAV routes planned by different algorithms in Figure 13 and Figure 14 with those in Figure 10 and Figure 11, it is evident that the paths generated by some inferior algorithms have exceeded the set constraint range and exhibit more turns and detours. In contrast, the MSDOA algorithm can still avoid obstacles and find the shortest, smoothest path, demonstrating its path planning capabilities. In addition, MSDOA demonstrated a 9% reduction in optimal fitness and a 12% reduction in average fitness compared to standard DOA in the most complex scenarios.

**Figure 15:**
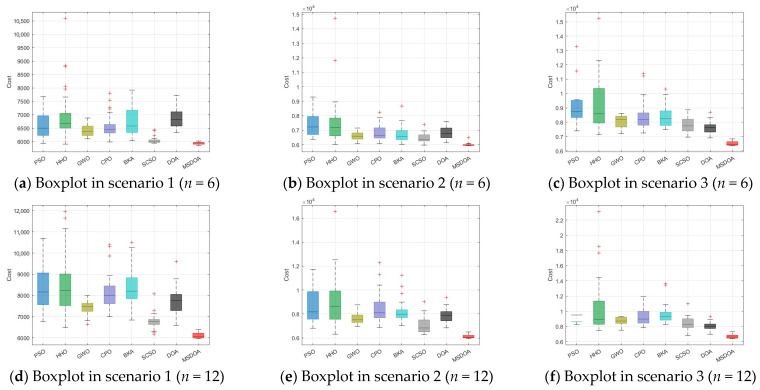
Boxplot comparison of path planning costs.

The box plot in Figure 15 illustrates that the MSDOA algorithm exhibits minimal cost fluctuations, indicative of its robust and scalable performance across complex environments. Figure 15a–c corresponds to scenarios with 6 waypoints, while Figure 15d–f represents scenarios with 12 waypoints. In both cases, the median cost of MSDOA is notably lower than that of the comparative algorithms, including PSO, HHO, and BKA. Additionally, the interquartile range of MSDOA remains consistently smaller, demonstrating its stability. Despite increasing scenario complexity or the number of waypoints, MSDOA exhibits significantly improved global search capabilities in complex optimization environments, attains superior optimization accuracy, and effectively mitigates the risk of converging to local optima. These findings confirm its efficacy in tackling UAV path planning challenges.

In conclusion, the MSDOA algorithm demonstrates robust convergence in addressing the three-dimensional path planning problem for drones in complex environments. Initially, it leverages a Bernoulli chaotic map to generate an improved initial solution. During exploration, adaptive perturbation and the Lens Imaging Learning Strategy facilitate rapid convergence to the optimal solution. Ultimately, the Adaptive Individual-level Mixed Strategy significantly enhances solution accuracy at final convergence, maintaining strong performance across diverse scenarios.

## 6. Conclusions

In this study, a multi-strategy MSDOA algorithm demonstrates robust performance in solving complex 3D path planning problems for UAVs. The algorithm employs a Bernoulli chaotic map to initialize the population, widen individual search ranges, and enhance population diversity. Furthermore, the algorithm incorporates an adaptive disturbance mechanism and a lens imaging reverse learning strategy during the exploration phase to update the population, thereby improving the exploration ability and accelerating convergence while mitigating premature convergence. Additionally, an Adaptive Individual-level Mixed Strategy (AIMS) is developed to conduct a more flexible search process and enhance the algorithm’s global search capability. To validate the effectiveness of these improvements, comparative experiments were conducted using the CEC 2017 benchmark functions. The results indicate that the MSDOA algorithm outperforms mainstream swarm intelligence algorithms in terms of convergence speed and optimal value search ability, particularly when addressing multi-peak, nonlinear, and high-dimensional optimization problems. Moreover, the MSDOA algorithm is applied to UAV 3D path planning problems under various scene models, and its performance is compared with other algorithms. The simulation results demonstrate that the MSDOA algorithm achieves more efficient and higher-quality path planning in complex 3D environments, resulting in smoother and safer UAV paths.

## Figures and Tables

**Table 1:** Comparison of optimization results of test functions.

Function	Index	MSDOA	DOA	PSO	HHO	GWO	CPO	BKA	SCSO
F1	Best	**2.31 × 10^3^**	3.54 × 10^5^	9.53 × 10^9^	1.16 × 10^8^	5.88 × 10^7^	2.44 × 10^8^	1.39 × 10^9^	4.81 × 10^9^
	Mean	**1.83 × 10** **^4^**	1.03 × 10^6^	1.98 × 10^10^	4.44 × 10^8^	2.63 × 10^9^	2.94 × 10^9^	6.71 × 10^9^	9.52 × 10^9^
	Std	**1.61 × 10** **^8^**	1.56 × 10^11^	4.43 × 10^19^	9.14 × 10^16^	2.62 × 10^18^	5.87 × 10^18^	1.05 × 10^19^	1.32 × 10^19^
F3	Best	**2.** **14 × 10^3^**	5.14 × 10^4^	2.49 × 10^4^	4.71 × 10^4^	4.03 × 10^4^	1.21 × 10^5^	1.45 × 10^4^	1.15 × 10^5^
	Mean	**8.71 × 10^3^**	1.15 × 10^5^	5.54 × 10^4^	5.79 × 10^4^	6.32 × 10^4^	2.21 × 10^5^	2.91 × 10^4^	1.81 × 10^5^
	Std	**1.78 × 10** **^7^**	8.78 × 10^8^	2.98 × 10^8^	5.22 × 10^7^	l.17 × 10^8^	4.39 × 10^9^	7.24 × 10^7^	8.51 × 10^8^
F4	Best	**4.03 × 10** **^2^**	4.72 × 10^2^	1.50 × 10^3^	5.84 × 10^2^	5.39 × 10^2^	5.59 × 10^2^	5.31 × 10^2^	8.8 × 10^2^
	Mean	**4.38 × 10** **^2^**	5.01 × 10^2^	4.55 × 10^3^	7.27 × 10^2^	6.63 × 10^2^	6.56 × 10^2^	9.64 × 10^2^	l.65 × 10^3^
	Std	**4.67 × 10** **^2^**	2.14 × 10^3^	3.54 × 10^6^	1.28 × 10^4^	1.14 × 10^4^	7.45 × 10^3^	1.28 × 10^5^	5.49 × 10^5^
F5	Best	**5.31 × 10** **^2^**	5.55 × 10^2^	7.15 × 10^2^	6.97 × 10^2^	5.88 × 10^2^	6.67 × 10^2^	6.96 × 10^2^	6.97 × 10^2^
	Mean	**5.60 × 10** **^2^**	5.81 × 10^2^	7.89 × 10^2^	7.7 × 10^2^	6.27 × 10^2^	7.57 × 10^2^	7.83 × 10^2^	7.98 × 10^2^
	Std	**2.37 × 10** **^2^**	2.63 × 10^2^	2.78 × 10^3^	7.91 × 10^2^	1.82 × 10^3^	2.54 × 10^3^	2.77 × 10^5^	3.02 × 10^2^
F6	Best	**6 × 10** **^2^**	6.32 × 10^2^	6.5 × 10^2^	6.61 × 10^2^	6.06 × 10^2^	6.47 × 10^2^	6.47 × 10^2^	6.14 × 10^2^
	Mean	**6 × 10** **^2^**	6.43 × 10^2^	6.72 × 10^2^	6.66 × 10^2^	6.13 × 10^2^	6.67 × 10^2^	6.61 × 10^2^	6.3 × 10^2^
	Std	**4.54 × 10** **^−3^**	4.54 × 10^1^	1.13 × 10^2^	6.18 × 10^1^	3.08 × 10^1^	1.62 × 10^2^	5.82 × 10^1^	1.19 × 10^2^
F7	Best	**7.66 × 10** **^2^**	7.91 × 10^2^	1.07 × 10^3^	1.17 × 10^3^	8.24 × 10^2^	1.04 × 10^3^	1.07 × 10^3^	1.16 × 10^3^
	Mean	**7.99 × 10** **^2^**	8.27 × 10^2^	1.24 × 10^3^	1.32 × 10^3^	9.08 × 10^2^	1.17 × 10^3^	1.24 × 10^3^	1.34 × 10^3^
	Std	**2.83 × 10** **^2^**	3.03 × 10^2^	7.56 × 10^3^	3.35 × 10^3^	3.1 × 10^3^	8.59 × 10^3^	2.72 × 10^4^	2 × 10^4^
F8	Best	**8.38 × 10** **^2^**	8.84 × 10^2^	9.31 × 10^2^	9.36 × 10^2^	8.62 × 10^2^	9.26 × 10^2^	9.42 × 10^2^	9.67 × 10^2^
	Mean	**8.65 × 10** **^2^**	9.12 × 10^2^	1.03 × 10^3^	9.85 × 10^2^	9.15 × 10^2^	9.96 × 10^2^	1.01 × 10^3^	1.08 × 10^2^
	Std	**2.39 × 10** **^2^**	2.73 × 10^2^	2.96 × 10^3^	6.45 × 10^2^	1.27 × 10^3^	1.65 × 10^2^	1.16 × 10^2^	1.82 × 10^2^
**Function**	**Index**	**MSDOA**	**DOA**	**PSO**	**HHO**	**GWO**	**CPO**	**BKA**	**SCSO**
F9	Best	**9.** **17 × 10** **^2^**	1.12 × 10^3^	3.89 × 10^3^	7.14 × 10^3^	1.34 × 10^3^	4.27 × 10^3^	3.67 × 10^3^	4.11 × 10^3^
	Mean	**l.** **18 × 10** **^3^**	l.81 × 10^3^	7.85 × 10^3^	8.8 × 10^3^	2.58 × 10^3^	7.51 × 10^3^	5.53 × 10^3^	1.06 × 10^4^
	Std	**1.56 × 10** **^5^**	2.13 × 10^5^	5.75 × 10^6^	7.89 × 10^5^	1.48 × 10^6^	5.05 × 10^5^	5.56 × 10^5^	l.6 × 10^7^
F10	Best	**2.52 × 10** **^3^**	2.9 × 10^3^	7.69 × 10^3^	4.74 × 10^3^	3.41 × 10^3^	4.41 × 10^3^	4.45 × 10^3^	7.15 × 10^3^
	Mean	**3.24 × 10** **^3^**	3.71 × 10^3^	9.17 × 10^3^	6.32 × 10^3^	4.68 × 10^3^	6.48 × 10^3^	5.47 × 10^3^	8.61 × 10^3^
	Std	**8.93 × 10** **^4^**	1.51 × 10^5^	4.74 × 10^5^	2.96 × 10^5^	8.1 × 10^5^	5.51 × 10^5^	3.19 × 10^5^	4.29 × 10^5^
F11	Best	**1.1** **1 × 10** **^3^**	1.17 × 10^3^	1.37 × 10^3^	1.31 × 10^3^	1.31 × 10^3^	1.43 × 10^3^	1.29 × 10^3^	2.87 × 10^3^
	Mean	**1.16 × 10** **^3^**	2.53 × 10^3^	3.84 × 10^3^	1.58 × 10^3^	2.59 × 10^3^	1.72 × 10^3^	1.66 × 10^3^	1.02 × 10^4^
	Std	**1.09 × 10** **^3^**	4.16 × 10^5^	9.68 × 10^6^	1.86 × 10^4^	1.09 × 10^6^	1.46 × 10^5^	2.66 × 10^5^	1.87 × 10^7^
F12	Best	**1.4** **1 × 10** **^5^**	2.91 × 10^5^	5.77 × 10^8^	4.47 × 10^6^	9.77 × 10^5^	6.81 × 10^6^	2.29 × 10^6^	3.77 × 10^7^
	Mean	**2.02 × 10** **^6^**	2.9 × 10^6^	3.13 × 10^9^	7.64 × 10^7^	l.2 × 10^8^	4.56 × 10^7^	8.05 × 10^7^	2.16 × 10^8^
	Std	**l.06 × 10** **^12^**	2.76 × 10^12^	5.25 × 10^18^	5.36 × 10^15^	2.52 × 10^16^	6.57 × 10^14^	1.95 × 10^16^	3.91 × 10^16^
F13	Best	**2.16 × 10** **^3^**	9.32 × 10^3^	1.33 × 10^5^	3.98 × 10^5^	5.15 × 10^4^	1.36 × 10^5^	6.72 × 10^4^	2.28 × 10^4^
	Mean	**l.45 × 10** **^4^**	2.46 × 10^4^	4.14 × 10^8^	4.2 × 10^6^	l.74 × 10^7^	1.21 × 10^7^	5.33 × 10^5^	2.05 × 10^7^
	Std	**l.4 × 10** **^8^**	2.25 × 10^8^	9.83 × 10^17^	3.06 × 10^14^	2.95 × 10^15^	2.57 × 10^15^	2.84 × 10^11^	2.02 × 10^15^
F14	Best	4.61 × 10^3^	2.61 × 10^4^	3.09 × 10^3^	2.82 × 10^4^	5.71 × 10^3^	4.63 × 10^4^	**1.** **94 × 10** **^3^**	3.2 × 10^4^
	Mean	1.64 × 10^5^	3.64 × 10^5^	3.89 × 10^5^	1.28 × 10^6^	5.11 × 10^5^	8.93 × 10^5^	**2.** **83 × 10** **^4^**	9.98 × 10^5^
	Std	2.61 × 10^10^	1.52 × 10^11^	1.12 × 10^12^	1.36 × 10^12^	3.92 × 10^11^	8.61 × 10^11^	**7.** **23 × 10** **^8^**	1.92 × 10^12^
F15	Best	**1.55 × 10** **^3^**	2.29 × 10^3^	6.42 × 10^3^	2.93 × 10^4^	1.48 × 10^4^	1.18 × 10^4^	1.71 × 10^4^	1.82 × 10^4^
	Mean	**5.14 × 10** **^3^**	6.87 × 10^3^	2 × 10^4^	l.24 × 10^5^	1.01 × 10^6^	2.07 × 10^5^	5.57 × 10^4^	3.6 × 10^5^
	Std	**1.14 × 10** **^7^**	3.62 × 10^7^	9.35 × 10^7^	7.39 × 10^9^	2.35 × 10^12^	2.44 × 10^11^	1.29 × 10^9^	1.24 × 10^12^
F16	Best	**1.75 × 10** **^3^**	1.96 × 10^3^	3.25 × 10^3^	2.85 × 10^3^	2.21 × 10^3^	2.59 × 10^3^	2.49 × 10^3^	2.72 × 10^3^
	Mean	**2.36 × 10** **^3^**	2.41 × 10^3^	4.23 × 10^3^	3.64 × 10^3^	2.62 × 10^3^	3.43 × 10^3^	3.16 × 10^3^	3.44 × 10^3^
	Std	**4.2** **2 × 10** **^4^**	5.46 × 10^4^	7.6 × 10^5^	3.29 × 10^5^	8.06 × 10^4^	1.35 × 10^5^	2.17 × 10^5^	1.81 × 10^5^
F17	Best	**1.64 × 10** **^3^**	1.76 × 10^3^	2.31 × 10^3^	2.15 × 10^3^	1.82 × 10^3^	2.03 × 10^3^	1.81 × 10^3^	1.98 × 10^3^
	Mean	**1.96 × 10** **^3^**	2.04 × 10^3^	3.23 × 10^3^	2.75 × 10^3^	2.06 × 10^3^	2.56 × 10^3^	2.51 × 10^3^	2.59 × 10^3^
	Std	**1.92 × 10** **^4^**	2.02 × 10^4^	6.62 × 10^5^	1.02 × 10^5^	3.29 × 10^4^	6.91 × 10^4^	7.81 × 10^4^	8.08 × 10^4^
F18	Best	**2.** **97 × 10** **^4^**	1.75 × 10^5^	1.01 × 10^5^	2.45 × 10^5^	6.88 × 10^4^	8.95 × 10^4^	4.23 × 10^4^	7.75 × 10^5^
	Mean	**4.38 × 10** **^5^**	7.22 × 10^5^	5.17 × 10^6^	5.08 × 10^6^	2.44 × 10^6^	3.11 × 10^6^	2.31 × 10^6^	1.24 × 10^7^
	Std	**1.03 × 10** **^11^**	2.95 × 10^11^	8.93 × 10^13^	4.23 × 10^13^	7.73 × 10^12^	1.15 × 10^13^	5.89 × 10^10^	3.06 × 10^14^
F19	Best	**1.** **72 × 10** **^3^**	2.09 × 10^3^	4.73 × 10^3^	1.43 × 10^5^	1.65 × 10^4^	2.69 × 10^4^	6.11 × 10^4^	4.92 × 10^4^
	Mean	**4.84 × 10** **^3^**	6.86 × 10^3^	2.49 × 10^5^	1.47 × 10^6^	2.4 × 10^6^	9.88 × 10^5^	4.67 × 10^5^	3.8 × 10^6^
	Std	**8.25 × 10** **^6^**	1.6 × 10^7^	1.11 × 10^11^	1.21 × 10^12^	1.77 × 10^13^	1.22 × 10^12^	1.94 × 10^13^	5.24 × 10^13^
F20	Best	**1.93 × 10** **^3^**	2.17 × 10^3^	2.45 × 10^3^	2.33 × 10^3^	2.23 × 10^3^	2.43 × 10^3^	2.18 × 10^3^	2.41 × 10^3^
	Mean	**2.** **12 × 10** **^3^**	2.37 × 10^3^	3.19 × 10^3^	2.84 × 10^3^	2.5 × 10^3^	2.81 × 10^3^	2.62 × 10^3^	2.96 × 10^3^
	Std	**1.84 × 10** ^4^	2.08 × 10^4^	7.84 × 10^4^	5.37 × 10^4^	3.52 × 10^4^	3.98 × 10^4^	4.57 × 10^4^	2.63 × 10^4^
F21	Best	**2.27 × 10** **^3^**	2.35 × 10^3^	2.49 × 10^3^	2.52 × 10^3^	2.35 × 10^3^	2.46 × 10^3^	2.51 × 10^3^	2.45 × 10^3^
	Mean	**2.** **34 × 10** **^3^**	2.6 × 10^3^	2.69 × 10^3^	2.6 × 10^3^	2.4 × 10^3^	2.54 × 10^3^	2.58 × 10^3^	2.56 × 10^3^
	Std	**2.** **25 × 10** **^2^**	2.52 × 10^2^	3.57 × 10^3^	1.93 × 10^3^	1.68 × 10^3^	2.36 × 10^3^	2.35 × 10^3^	1.69 × 10^3^
F22	Best	**2.31 × 10** **^3^**	2.45 × 10^3^	6.25 × 10^3^	3.07 × 10^3^	2.51 × 10^3^	2.46 × 10^3^	3.01 × 10^3^	3.23 × 10^3^
	Mean	**3.65 × 10** **^3^**	4.82 × 10^3^	9.49 × 10^3^	6.88 × 10^3^	6.19 × 10^3^	7.41 × 10^3^	6.52 × 10^3^	7.85 × 10^3^
	Std	**1.55 × 10** **^6^**	2.55 × 10^6^	2.59 × 10^6^	2.49 × 10^6^	6.9 × 10^6^	3.82 × 10^6^	1.15 × 10^6^	6.06 × 10^6^
F23	Best	**2.43 × 10** **^3^**	2.69 × 10^3^	3.26 × 10^3^	3.11 × 10^3^	2.78 × 10^3^	2.84 × 10^3^	2.96 × 10^3^	2.83 × 10^3^
	Mean	**2.** **53 × 10** **^3^**	2.72 × 10^3^	3.76 × 10^3^	3.31 × 10^3^	2.8 × 10^3^	2.96 × 10^3^	3.16 × 10^3^	2.9 × 10^3^
	Std	**2.43 × 10** **^3^**	2.98 × 10^3^	9.42 × 10^3^	1.43 × 10^4^	4.65 × 10^3^	6.26 × 10^4^	2.14 × 10^3^	1.87 × 10^3^
F24	Best	**2.85 × 10** **^3^**	2.92 × 10^3^	3.51 × 10^3^	3.26 × 10^3^	2.96 × 10^3^	2.97 × 10^3^	3.02 × 10^3^	3.02 × 10^3^
	Mean	**2.93 × 10** **^3^**	2.98 × 10^3^	3.86 × 10^3^	3.51 × 10^3^	3.06 × 10^3^	3.11 × 10^3^	3.31 × 10^3^	3.08 × 10^3^
	Std	**1.01 × 10** **^3^**	1.53 × 10^3^	6.35 × 10^4^	2.75 × 10^4^	5.17 × 10^3^	5.93 × 10^3^	1.38 × 10^4^	1.66 × 10^3^
**Function**	**Index**	**MSDOA**	**DOA**	**PSO**	**HHO**	**GWO**	**CPO**	**BKA**	**SCSO**
F25	Best	**2.** **67 × 10** **^3^**	2.99 × 10^3^	3.13 × 10^3^	2.92 × 10^3^	2.95 × 10^3^	2.94 × 10^3^	2.95 × 10^3^	3.1 × 10^3^
	Mean	**2.88 × 10** **^3^**	3.05 × 10^3^	3.5 × 10^3^	3.02 × 10^3^	3.04 × 10^3^	3.01 × 10^3^	3.08 × 10^3^	3.65 × 10^3^
	Std	**6.1 × 10** **^1^**	1.21 × 10^3^	8.6 × 10^4^	1.27 × 10^3^	5.73 × 10^3^	3.22 × 10^3^	5.86 × 10^4^	1.82 × 10^5^
F26	Best	**2.8 × 10** **^3^**	3.43 × 10^3^	6.68 × 10^3^	3.13 × 10^3^	3.42 × 10^3^	3.35 × 10^3^	3.65 × 10^3^	5.87 × 10^3^
	Mean	**3.** **28 × 10** **^3^**	3.85 × 10^3^	9.47 × 10^3^	7.79 × 10^3^	5.02 × 10^3^	6.68 × 10^3^	7.63 × 10^3^	6.47 × 10^3^
	Std	**2.71 × 10** **^5^**	6.06 × 10^5^	1.06 × 10^6^	2.61 × 10^6^	3.09 × 10^5^	1.29 × 10^6^	2.85 × 10^6^	1.93 × 10^5^
F27	Best	**3.09 × 10** **^3^**	3.28 × 10^3^	3.81 × 10^3^	3.33 × 10^3^	3.23 × 10^3^	3.25 × 10^3^	3.25 × 10^3^	3.22 × 10^3^
	Mean	**3.2** **1 × 10** **^3^**	3.32 × 10^3^	4.73 × 10^3^	3.59 × 10^3^	3.27 × 10^3^	3.36 × 10^3^	3.44 × 10^3^	3.26 × 10^3^
	Std	**4.72 × 10** **^−8^**	8.96 × 10^1^	2.84 × 10^5^	4.01 × 10^4^	9.43 × 10^2^	7.28 × 10^3^	3.14 × 10^4^	5.65 × 10^2^
F28	Best	**3.** **18 × 10** **^3^**	3.34 × 10^3^	3.89 × 10^3^	3.31 × 10^3^	3.34 × 10^3^	6.32 × 10^3^	5.88 × 10^3^	3.63 × 10^3^
	Mean	**3.29 × 10** **^3^**	3.42 × 10^3^	4.74 × 10^3^	3.49 × 10^3^	3.51 × 10^3^	7.67 × 10^3^	6.53 × 10^3^	4.61 × 10^3^
	Std	**1.14 × 10** **^1^**	2.44 × 10^2^	2.43 × 10^5^	9.62 × 10^3^	2.75 × 10^4^	3.99 × 10^5^	8.87 × 10^4^	5.49 × 10^5^
F29	Best	**3.08 × 10** **^3^**	3.44 × 10^3^	4.77 × 10^3^	4.08 × 10^3^	3.54 × 10^4^	4.08 × 10^3^	3.85 × 10^3^	3.82 × 10^3^
	Mean	**3.46 × 10** **^3^**	3.62 × 10^3^	6.05 × 10^3^	5.04 × 10^3^	3.99 × 10^3^	4.96 × 10^3^	4.75 × 10^3^	4.53 × 10^3^
	Std	**1.** **57 × 10** **^4^**	2.16 × 10^4^	7.67 × 10^5^	2.92 × 10^5^	3.13 × 10^4^	2.23 × 10^5^	2.77 × 10^5^	7.6 × 10^6^
F30	Best	**3.** **23 × 10** **^3^**	1.05 × 10^4^	3.43 × 10^6^	1.23 × 10^6^	1.98 × 10^6^	5.35 × 10^5^	7.06 × 10^5^	2.23 × 10^4^
	Mean	**4.69 × 10** **^3^**	4.13 × 10^4^	1.43 × 10^8^	1.46 × 10^7^	1.49 × 10^7^	4.74 × 10^6^	3.81 × 10^6^	1.05 × 10^6^
	Std	**1.2 × 10** **^6^**	1.66 × 10^9^	4.24 × 10^6^	1.08 × 10^15^	1.23 × 10^14^	1.47 × 10^13^	6.26 × 10^12^	1.85 × 10^12^

**Table 2:** Scenario information.

Scenario Number	Threat Center	Threat Radius	Threat Height
1	(300, 300)	50	230
	(700, 300)	50	230
	(500, 600)	60	250
2	(300, 280)	50	220
	(700, 280)	45	230
	(300, 520)	50	240
	(700, 520)	45	250
	(500, 400)	60	260
	(500, 580)	50	240
3	(320, 280)	40	130
	(480, 300)	45	135
	(620, 260)	40	125
	(350, 420)	50	140
	(520, 480)	60	300
	(660, 420)	50	140
	(370, 600)	45	200
	(500, 640)	45	135
	(620, 590)	40	200

**Table 3:** Complex scenario simulation experiment data (number of waypoints = 6).

Scenario	Index	MSDOA	DOA	PSO	HHO	GWO	CPO	BKA	SCSO
Scenario 1	Best	**5.82 × 10** **^3^**	6.29 × 10^3^	5.93 × 10^3^	5.91 × 10^3^	6.11 × 10^3^	5.98 × 10^3^	6.03 × 10^3^	5.98 × 10^3^
	Mean	**5.9** **1 × 10** **^3^**	7.01 × 10^3^	7.47 × 10^3^	7.26 × 10^3^	6.89 × 10^3^	7.48 × 10^3^	7.24 × 10^4^	6.21 × 10^3^
	Std	**4.4 × 10** **^1^**	3.72 × 10^2^	1.06 × 10^3^	1.53 × 10^2^	4.76 × 10^2^	8.31 × 10^2^	7.91 × 10^2^	1.2 × 10^2^
Scenario 2	Best	**5.94 × 10** **^3^**	6.41 × 10^3^	6.74 × 10^3^	6.68 × 10^3^	6.55 × 10^3^	6.39 × 10^3^	6.47 × 10^3^	6.19 × 10^3^
	Mean	**5.99 × 10** **^3^**	7.21 × 10^3^	7.87 × 10^3^	7.78 × 10^4^	7.01 × 10^3^	7.87 × 10^3^	7.37 × 10^3^	6.53 × 10^4^
	Std	**1.02 × 10** **^2^**	6.12 × 10^2^	1.84 × 10^3^	1.76 × 10^3^	7.12 × 10^2^	8.75 × 10^2^	8.87 × 10^2^	4.53 × 10^2^
Scenario 3	Best	**6.37 × 10** **^3^**	6.91 × 10^3^	7.35 × 10^3^	7.13 × 10^3^	7.21 × 10^3^	7.25 × 10^3^	7.51 × 10^3^	6.96 × 10^3^
	Mean	**6.** **52 × 10** **^3^**	7.65 × 10^3^	8.96 × 10^3^	9.35 × 10^3^	8.39 × 10^3^	8.41 × 10^3^	8.37 × 10^4^	7.77 × 10^3^
	Std	**1.** **63 × 10** **^2^**	4.28 × 10^2^	2.14 × 10^3^	1.84 × 10^3^	1.04 × 10^3^	1.09 × 10^2^	9.53 × 10^3^	5.11 × 10^2^

**Table 4:** Complex scenario simulation experiment data (number of waypoints = 12).

Scenario	Index	MSDOA	DOA	PSO	HHO	GWO	CPO	BKA	SCSO
Scenario 1	Best	**5.95 × 10** **^3^**	6.58 × 10^3^	6.78 × 10^3^	6.48 × 10^3^	6.64 × 10^3^	6.99 × 10^3^	6.83 × 10^3^	6.17 × 10^3^
	Mean	**6.** **12 × 10** **^3^**	7.76 × 10^3^	8.38 × 10^3^	8.44 × 10^3^	7.43 × 10^3^	8.2 × 10^3^	8.35 × 10^4^	6.76 × 10^3^
	Std	**1.37 × 10** **^2^**	5.95 × 10^2^	1.46 × 10^3^	1.41 × 10^2^	5.82 × 10^2^	8.21 × 10^2^	9.14 × 10^2^	3.57 × 10^2^
Scenario 2	Best	**5.91 × 10** **^3^**	6.83 × 10^3^	6.81 × 10^3^	6.57 × 10^3^	6.96 × 10^3^	6.87 × 10^3^	7.02 × 10^3^	6.38 × 10^3^
	Mean	**6.11 × 10** **^3^**	7.89 × 10^3^	8.15 × 10^4^	9.06 × 10^3^	7.51 × 10^3^	8.48 × 10^3^	8.56 × 10^3^	7.04 × 10^4^
	Std	**1.59 × 10** **^2^**	5.38 × 10^2^	2.84 × 10^3^	2.21 × 10^3^	9.85 × 10^2^	1.18 × 10	9.12 × 10^2^	6.59 × 10^3^
Scenario 3	Best	**6.39 × 10** **^3^**	6.97 × 10^3^	8.25 × 10^3^	7.48 × 10^3^	7.51 × 10^3^	7.85 × 10^3^	8.25 × 10^3^	6.81 × 10^3^
	Mean	**6.** **68 × 10** **^3^**	8.05 × 10^3^	1.06 × 10^3^	1.05 × 10^4^	1.09 × 10^3^	9.22 × 10^3^	9.59 × 10^4^	8.34 × 10^3^
	Std	**2.25 × 10** **^2^**	6.06 × 10^2^	3.14 × 10^3^	3.66 × 10^3^	4.04 × 10^3^	1.33 × 10^2^	1.23 × 10^2^	8.37 × 10^2^

## Data Availability

Available under request.
